# Temporal Patterns of Holter-Detected Arrhythmias in Hypertrophic Cardiomyopathy Patients Treated with Mavacamten

**DOI:** 10.3390/biomedicines13041005

**Published:** 2025-04-21

**Authors:** Amro Badr, Kaitlin Roehl, Mustafa Suppah, Humam Abo Abdullah, Reza Arsanjani, Konstantinos C. Siontis, Jeffrey B. Geske, Steve R. Ommen, John R. Giudicessi, Said Alsidawi

**Affiliations:** 1 Department of Cardiovascular Medicine, Mayo Clinic, Phoenix, AZ 85054, USA; badr.amro@mayo.edu (A.B.); roehl.kaitlin@mayo.edu (K.R.); suppah.mustafa@mayo.edu (M.S.); aboabdullah.humam@mayo.edu (H.A.A.); arsanjani.reza@mayo.edu (R.A.); 2 Department of Cardiovascular Medicine, Mayo Clinic, Rochester, MN 55905, USA; siontis.konstantinos@mayo.edu (K.C.S.); geske.jeffrey@mayo.edu (J.B.G.); ommen.steve@mayo.edu (S.R.O.); giudicessi.john@mayo.edu (J.R.G.)

**Keywords:** mavacamten, HCM, cardiomyopathy, arrythmia, Holter

## Abstract

**Background:** Hypertrophic cardiomyopathy (HCM) is a genetic cardiomyopathy marked by increased left ventricular wall thickness, leading in some cases to left ventricular outflow tract (LVOT) obstruction, heart failure, and arrhythmias. Mavacamten, a selective allosteric inhibitor of cardiac myosin, has demonstrated benefits in improving hemodynamics and reducing LVOT obstruction. However, its impact on arrhythmic burden remains unclear, with reports of early atrial fibrillation (AF) risk contrasting with long-term reductions in arrhythmias. This study assesses the temporal patterns of Holter-detected arrhythmias in HCM patients treated with mavacamten. **Methods:** This retrospective study included HCM patients from three Mayo Clinic sites. Baseline demographic, clinical, and echocardiographic data were collected. Holter monitoring was performed at baseline, short-term (<6 months), and long-term (>6 months) follow-up. Arrhythmic events, including premature atrial contractions (PACs), premature ventricular contractions (PVCs), and supraventricular tachycardia (SVT), were analyzed using standardized rates per 24 h. Statistical comparisons utilized the Wilcoxon signed-rank test. **Results:** Twenty-seven patients (56% female, median age 66 years) were included. PACs, PVCs, and SVT duration transiently but not significantly increased at short-term follow-up but returned to baseline at long-term follow-up. No sustained or high-risk ventricular arrhythmias were observed. **Conclusions:** Mavacamten is associated with transient arrhythmic fluctuations early in treatment, followed by stabilization. These findings support its long-term electrophysiological safety and underscore the need for early rhythm monitoring. Further research should explore its role in arrhythmic risk stratification in HCM patients.

## 1. Introduction

Hypertrophic cardiomyopathy (HCM) is the most common genetic heart disease characterized by unexplained increased left ventricular wall thickness not attributable to overloading conditions such as hypertension and aortic stenosis [1]. HCM is estimated to affect 1 in every 500 adults worldwide and is primarily caused by mutations in sarcomere protein genes encoding for components of the contractile apparatus of the heart [2]. These mutations display different patterns of inheritance, with autosomal dominance being the predominant form. Approximately 50–60% of diagnosed cases are linked to identifiable familial disease [2].

The wall thickening impairs the heart’s ability to pump blood effectively and can cause obstruction of the left ventricular outflow tract (LVOT). This can often lead to symptoms of exertional dyspnea, reduced exercise capacity, and syncope. HCM is also associated with mitral regurgitation, diastolic dysfunction, interstitial fibrosis, myocyte disarray, and myocardial ischemia. As a result, patients with HCM have an increased risk of heart failure, arrhythmias, and sudden cardiac death [3,4]. Non-sustained ventricular tachycardia (NSVT) is present in 20% to 30% of patients with HCM and can lead to ventricular fibrillation, which is the most common cause of sudden cardiac death [5].

Premature atrial contractions (PACs) are precursors to atrial fibrillation (AF), especially in individuals with left atrial enlargement and impaired function [6]. In hypertrophic cardiomyopathy (HCM), left atrial remodeling, stemming from diastolic dysfunction and elevated filling pressures, increases the likelihood of AF [7]. Frequent PACs, particularly those with short coupling intervals, have been linked to a heightened risk of AF onset, a critical prognostic factor in HCM due to its association with thromboembolism and heart failure progression [6,8,9].

While isolated premature ventricular contractions (PVCs) are often benign, frequent, or complex PVCs may suggest an elevated risk of malignant arrhythmias in HCM [10,11]. Extensive myocardial fibrosis, identifiable through late gadolinium enhancement on cardiac MRI, is associated with increased risk of ventricular tachycardia (VT) and sudden cardiac death (SCD) [12,13]. PVC burden can serve as an indicator of arrhythmogenic substrate in HCM patients [14].

Current management strategies for HCM encompass lifestyle modifications, familial screening with genetic counseling, and pharmacotherapy to manage symptoms [15]. Guideline-directed therapy involves non-selective drugs like beta-blockers, non-dihydropyridine calcium channel blockers, and disopyramide, primarily for individuals experiencing symptomatic obstruction of the outflow tract [16]. Beta-blockers, often the first-line treatment for relieving left ventricular outflow tract obstruction (LVOTO), reduce heart rate and cardiac muscle contraction force, alleviating symptoms and effectively reducing exercise-induced LVOTO [17,18].

Beta-blockers can modestly alleviate symptoms in HCM patients, though their impact on ventricular arrhythmias or sudden cardiac death is less established [11,19,20]. Non-dihydropyridine calcium channel blockers, such as diltiazem or verapamil, serve as antiarrhythmic agents, especially for supraventricular arrhythmias, and are recommended for HCM patients who do not tolerate beta-blockers well [16,21]. These calcium channel blockers have negative inotropic and chronotropic effects, aiding in the management of HCM symptoms [22].

Disopyramide improves clinical symptoms, however it has not been associated with improved survival or reduced cardiovascular deaths, including sudden arrhythmic death [23,24]. Studies indicate that disopyramide reduces LVOT gradients, slightly decreases resting ejection fraction, and modestly increases the corrected QT interval [23]. A study showed that disopyramide prolonged QTc, QRS, JT, and PR intervals without significantly changing heart rate [24]. The drug’s effects on cellular arrhythmia triggers, like early afterdepolarizations (EADs) and delayed afterdepolarizations (DADs), can be beneficial [25].

Mavacamten is a novel selective allosteric inhibitor of cardiac-specific myosin that reversibly inhibits actin–myosin cross-bridging. Mavacamten acts by normalizing the force of contraction and alleviating LVOT obstruction, thus improving blood flow and reducing symptoms associated with the disease [26]. Previous studies of patients with obstructive HCM showed that mavacamten significantly reduced LVOT gradient and improved symptoms and quality of life [27,28,29].

Studies have also demonstrated that mavacamten promotes favorable cardiac remodeling, including improvements in LAVI, LV diastolic function, and biomarkers such as N-terminal pro-B-type natriuretic peptide (NT-proBNP) and troponin [30,31]. However, despite these structural improvements, its effect on left atrial (LA) function in patients with pre-existing atrial fibrillation (AF) remains limited, with no significant changes observed in LA conduit, contraction, or reservoir strain [32].

In addition to mavacamten’s structural effects, it has been associated with improved electromechanical dispersion, including reductions in QTc dispersion and peak systolic dispersion as assessed by speckle tracking analysis. These changes suggest reduced electromechanical dispersion, a critical determinant of rhythm stability and a predictor of arrhythmias [33].

While these structural and functional changes theoretically reduce the arrhythmia substrate and thus should reduce arrhythmia burden, current evidence on mavacamten’s impact on the incidence of atrial or ventricular arrhythmias remains heterogenous. Some studies suggest no significant effect on arrythmia incidence, while others indicate an increase in new-onset atrial fibrillation during the first few months of therapy [34].

In this study, we aim to address the critical gap in understanding the electrophysiological effects of mavacamten in HCM patients by using Holter monitoring to capture detailed and continuous cardiac rhythm data and provide a comprehensive assessment of arrhythmia burden over an extended period, enabling the detection of transient and asymptomatic arrhythmias that might not be detected otherwise.

## 2. Methods

This is a retrospective study involving HCM patients treated with mavacamten who had clinically indicated Holter monitoring before and after starting mavacamten at three tertiary hospitals in the United States (Mayo Clinic Rochester, MN, USA, Mayo Clinic Arizona, AZ, USA, Mayo Clinic Florida, FL, USA). We included patients with hypertrophic cardiomyopathy and baseline New York Heart Association (NYHA) functional classification of two and three. Patients were excluded if they had (1) unstable arrhythmia burden at baseline with recent worsening, (2) major clinical interventions during the study period (e.g., ablations, device changes, new antiarrhythmic medications), or (3) cardiovascular events that could significantly impact arrhythmia burden during follow-up such as myocardial infarction and decompensated heart failure. Eligible patients were initiated on a starting dose of 5 mg of mavacamten daily per mavacamten established protocol. Dosage adjustments were guided by periodic echocardiographic assessments to achieve two primary goals: reduction in the left ventricular outflow tract (LVOT) gradient to <30 mmHg and maintenance of the left ventricular ejection fraction (LVEF) ≥ 55%. Titration intervals were determined by the clinical judgment of treating physicians in accordance with established protocols.

The sample size was calculated by G*power software (version 3.1.9.7). A priori power analysis was conducted for the Wilcoxon signed-rank test, assuming a large effect size (Cohen’s d = 0.8) with a power of 80% and a significance level of 5% (α = 0.05). The minimum sample required to detect a statistically significant difference was determined to be 15 subjects.

We reviewed patients’ electronic medical charts to collect baseline demographic and clinical characteristics. Additionally, we analyzed their echocardiographic studies prior to initiating mavacamten to establish baseline echocardiographic parameters including LVOT gradient, ejection fraction, and left ventricular wall thickness. We extracted data on the burden of premature ventricular contractions (PVCs), premature atrial contractions (PACs), as well as the presence and duration supraventricular tachycardia (SVT) at three different time points: baseline Holter, short-term follow-up Holter within 6 months after initiating mavacamten, and longer-term follow-up Holter beyond 6 months using the MoMe Wearable cardiac monitor. To account for the varying lengths of Holter monitoring among patients, we standardized the data extracted from Holter monitoring by calculating the arrhythmia rates per 24 h for each patient.

Continuous variables were descriptively represented using median and interquartile range (IQR), and statistical comparisons between different time points were performed utilizing the Wilcoxon signed-rank test. Statistical significance was defined as a *p*-value of less than 0.05, and all statistical procedures were performed using R statistical packages (version 4.4.2).

## 3. Results

Twenty-seven patients were included in the study, including 15 (56%) females. The median age was 66 (IQR 58–72) years. Hypertension was the most prevalent comorbidity, observed in 70% of the cohort, followed by atrial fibrillation (AF), present in 30%. Nearly half of the patients (48%) had an implantable cardioverter defibrillator (ICD). Chronic kidney disease (CKD) and diabetes mellitus were noted in 18.5% and 11% of the participants, respectively (Table 1).

Beta-blockers were the most prescribed therapy, used by 85% of the cohort. In contrast, calcium channel blockers and antiarrhythmic agents were less frequently utilized, prescribed in 11% and 3% of patients, respectively (Table 1).

The echocardiographic characteristics of the cohort are shown in (Table 1). The ejection fraction was preserved in all participants with a median of 71% (IQR: 66.5–73.5). The median left ventricular outflow tract (LVOT) gradient was 52 mmHg (IQR: 17.5–101) at rest. The median left ventricular (LV) mass index was 126.5 g/m^2^ (IQR: 103.74–135.5), with a maximum LV wall thickness of 19 mm (IQR: 17–21) and a median LV septal thickness of 15 mm (IQR: 13–17).

All patients had at least two Holter monitoring sessions, with seven having three sessions. The median duration between baseline and short-term Holter was 4 (IQR 2–4.5) months, while the median duration to the most recent Holter was 11 (IQR 9–13.5) months. The median time on mavacamten until short-term Holter was 4 (IQR 2–4) months and until long-term Holter was 11 (IQR 11–18) months.

At the short-term follow-up, the number of PVCs remained similar to baseline [19 (IQR 7–494) vs. 24 (IQR 2–286), *p* = 0.63]. The number of PACs also showed no significant difference [145 (IQR 28–622) vs. 70 (IQR 16–231), *p* = 0.69]. There were no changes in the number of SVT runs [0.76 (IQR 0–4.00) vs. 0.25 (IQR 0–3.13), *p* = 0.67] or SVT max duration [6 (IQR 0–19) vs. 2 (IQR 0–10), *p* = 0.09]. Similarly, SVT max rate remained unchanged between baseline and short-term follow-up [115 (IQR 0–152) vs. 48 (IQR 0–130), *p* = 0.44] (Table 2).

**Figure 1:**
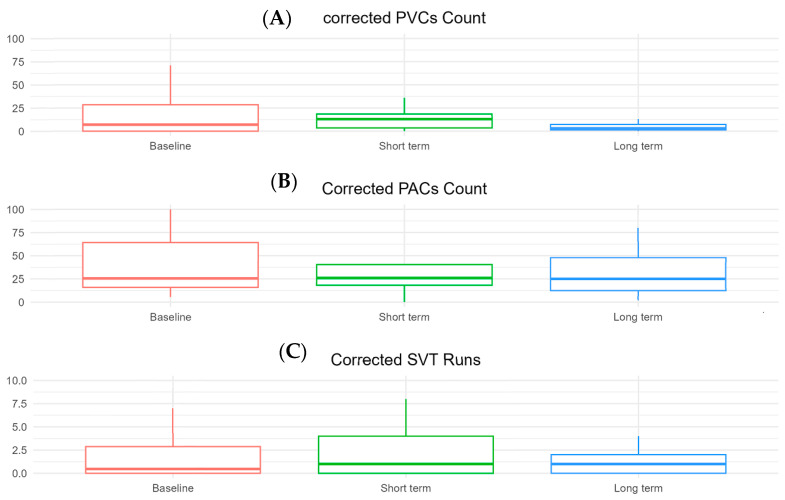
Boxplots comparing key Holter-detected arrhythmias in patients on mavacamten between baseline and long-term follow-up. (**A**) Corrected premature ventricular complexes count, (**B**) corrected premature atrial complexes count, (**C**) supraventricular tachycardia runs.

At the long-term follow-up (Figure 1), the number of PVCs remained similar to baseline [5 (IQR 1–40) vs. 13 (IQR 1–39), *p* = 0.85]. The number of PACs did not change significantly [53 (IQR 18–204) vs. 75 (IQR 24–166), *p* = 0.37]. There were no significant differences in the number of SVT runs [1.00 (IQR 0–2.00) vs. 0.47 (IQR 0–3.00), *p* = 0.27] or in SVT max duration [3 (IQR 0–9) vs. 3 (IQR 0–10), *p* = 0.46]. The SVT max rate also remained unchanged [109 (IQR 0–142) vs. 102 (IQR 0–141), *p* = 0.90] (Table 3).

## 4. Discussion

Our study provides preliminary insights into the temporal patterns of arrhythmias in hypertrophic cardiomyopathy (HCM) patients treated with mavacamten, using Holter monitoring over an extended follow-up period. The key findings indicate a transient but non-significant increase in premature atrial contractions (PACs), and supraventricular tachycardia (SVT) duration shortly after mavacamten initiation, followed by a return to baseline levels at long-term follow-up. These findings suggest that early electrophysiological fluctuations occur but do not translate into a sustained arrhythmic burden. Our results expand on previous studies that have discussed the benefits of mavacamten in reducing left ventricular outflow tract (LVOT) gradients, improving diastolic function, and enhancing the quality of life in patients with symptomatic obstructive HCM [4,28,35,36,37,38]

Our findings build upon prior research on the electrophysiological effects of mavacamten. Previous studies, such as the EXPLORER-HCM trial, demonstrated significant hemodynamic and symptomatic benefits of mavacamten in patients with obstructive HCM, highlighting its role in reducing LVOT obstruction and improving left atrial function [28]. The impact of mavacamten on atrial arrhythmias in hypertrophic obstructive cardiomyopathy (HOCM) remains debated. Some studies suggest an increased incidence of atrial fibrillation (AF) early in treatment, likely due to acute hemodynamic shifts or autonomic modulation [35]. Conversely, long-term data indicate that mavacamten enhances left atrial function and remodeling, which may ultimately reduce AF risk [33,36].

Our findings bridge this discrepancy, showing that while PACs and SVT duration initially slightly rise, they later stabilize, suggesting a transient electrophysiological adaptation rather than a sustained proarrhythmic effect. This aligns with evidence that early hemodynamic changes may momentarily disrupt atrial electrophysiology before leading to structural improvements and reduced arrhythmogenicity [33,36,39,40]. Supporting this, prior studies demonstrate that mavacamten lowers left atrial volume index (LAVI) and myocardial stress biomarkers, both linked to AF risk [39], while prolonged therapy enhances left atrial strain and function [30].

While initial studies highlight an early AF risk [34], longer-term analyses suggest these transient effects are offset by sustained improvements in left atrial function [30,32,36]. Longitudinal data indicate that mavacamten enhances left atrial reservoir strain, potentially reducing AF progression [33], while a retrospective study links it to reduced HCM phenotype severity and improved AI-ECG markers of AF risk. AI-ECG analysis further suggests mavacamten decreases AF probability while improving ST-T segment abnormalities [36]. Emerging evidence also indicates mavacamten may lower the HCM-AF score by improving atrial structure and function, with AI-ECG showing a significant reduction in AF risk after 12 weeks of treatment [37].

The transient slight and non-significant increase in PACs and SVT duration observed in the early months of therapy may be attributed to several physiological mechanisms. First, as mavacamten rapidly reduces LVOT gradients, there may be acute alterations in left atrial pressure and preload, leading to transient atrial and ventricular ectopy [3]. Second, many patients initiating mavacamten undergo tapering or discontinuation of beta-blockers, which could modulate autonomic tone and influence arrhythmic susceptibility [41]. Third, the improvements in the LV mass index and diastolic function associated with mavacamten therapy may contribute to the long-term stabilization of atrial electrical activity, thereby reducing PAC burden over time [39]. Despite these transient arrhythmic changes, no sustained or high-risk ventricular arrhythmias were observed, reinforcing the overall safety profile of mavacamten in terms of electrophysiological stability [4].

These findings have important clinical implications for managing HCM patients on mavacamten. Given the transient arrhythmic increase observed early in treatment [34], clinicians should consider routine short-term Holter monitoring within three to six months of initiation, particularly in patients with a prior history of AF or other atrial arrythmias. Additionally, although our findings provide some reassurance regarding the long-term safety profile of mavacamten, as it suggests that mavacamten does not inherently increase arrhythmic risk, alleviating concerns about its long-term proarrhythmic potential, given the exploratory nature of our study due to the retrospective design as well as the small sample size, longitudinal follow-up is still necessary to determine if the observed arrhythmic fluctuations impact long-term arrhythmic outcomes or survival rate. This can be particularly relevant in patients with ICDs or those with pre-existing atrial arrhythmias who may require individualized arrhythmic risk assessment and closer follow-up [29]. While mavacamten will alter the risk of ventricular arrythmias, sudden cardiac death and the overall scan burden in patients with HCM is yet to be determined through long-term data follow-up.

A few limitations we encountered must be acknowledged. The study’s small sample size of 27 patients may not be fully powered to detect rare but clinically significant arrhythmic events. Additionally, variability in Holter monitoring durations across patients could have influenced arrhythmia detection rates, although standardized adjustments were made. Another key limitation is the absence of a matched control cohort, making it unclear whether the observed patterns are specific to mavacamten or reflect natural arrhythmic fluctuations in HCM patients. Future research should aim to address these gaps by utilizing larger, prospective studies with longer follow-up periods and incorporating more rigorous monitoring techniques, such as implantable loop recorders, to capture subclinical arrhythmias.

Despite our findings supporting the electrophysiological safety profile of mavacamten, we have not yet explored its impact when used in combination with other HCM therapies, such as beta-blockers, calcium channel blockers, and antiarrhythmic agents. This presents an opportunity for future research to better understand the safety and efficacy of these combination therapies. To further elucidate the electrophysiological effects of mavacamten, future studies should focus on utilizing prolonged monitoring strategies, such as implantable loop recorders, which provide continuous long-term rhythm assessment and capture subclinical arrhythmias. HCM is predominantly an inherited disorder, often resulting from mutations in genes encoding sarcomeric proteins, notably *MYH7* and *MYBPC3*. These mutations lead to diverse clinical manifestations, influencing disease severity, progression, and arrhythmic risk. Advancements in cardio-genomics have enhanced our understanding of HCM’s genetic underpinnings. Genetic testing facilitates early diagnosis, family screening, and personalized management strategies [38,42]. The heterogeneity in genetic mutations contributes to the variability observed in clinical outcomes among HCM patients. Therefore, future studies should also explore patient-specific factors that may influence arrhythmic responses to mavacamten, including genetic variants, comorbid conditions, and baseline electrophysiological characteristics. Stratification by genetic mutations, such as *MYH7*, *MYBPC3*, and other sarcomere gene mutations, could also provide insights into the genotype–phenotype correlations, enabling personalized treatment approaches [38,42]. Additionally, exploring the influence of pre-existing atrial arrythmias, atrial fibrosis burden, and left atrial structural remodeling could help identify high-risk subgroups. This approach would allow for better risk stratification and the development of individualized therapeutic strategies, potentially optimizing outcomes for specific patient populations.

## 5. Conclusions

Our study provides a foundation for future research aiming to assess the role of mavacamten in HCM patients by evaluating its arrhythmic burden. Our findings highlight a transient fluctuation in PACs, PVCs, and SVT duration early in treatment, but these changes were not statistically significant and returned to baseline at long-term follow-up.

### Clinical Significance

These findings reinforce the long-term electrophysiological safety of mavacamten. This is currently an area of debate and there are concerns about the early arrhythmogenic potential of mavacamten. The study underscores the importance of early rhythm monitoring while providing reassurance regarding the overall arrhythmic profile of the drug. These results contribute to the growing body of evidence investigating mavacamten’s safety and efficacy in HCM management, suggesting an overall very safe profile. It is reasonable for clinicians, however, to closely monitor patients at risk of arrhythmias (known history of atrial fibrillation, high burden of atrial or ventricular ectopy) after initiating mavacamten. Given the small sample size and retrospective design of our study, these findings should be explored in future research with larger, prolonged studies to further characterize its impact on cardiac arrhythmia and optimize patient management strategies.

## Figures and Tables

**Table 1:** Baseline clinical and echocardiographic characteristics of the study population.

Characteristic	*n* = 27
**Demographics**	
Age, years (median [IQR])	66 [58–72]
Age group, *n* (%)	
<50	2 (7%)
50–70	13 (48%)
>70	10 (37%)
Sex	
Male	12 (35%)
Female	15 (65%)
**Clinical characteristics**	
Hypertension, *n* (%)	19 (70%)
Diabetes mellitus, *n* (%)	3 (11%)
CKD, *n* (%)	5 (18.5)
Asthma/COPD, *n* (%)	6 (22%)
Afib, *n* (%)	8 (30%)
Afib ablation, *n* (%)	2 (7%)
ICD Implantation, *n* (%)	13 (48%)
**Medications**	
Beta-blockers, *n* (%)	23 (85%)
Calcium channel blockers, *n* (%)	3 (11%)
Antiarrhythmics, *n* (%)	1 (3%)
**Echocardiographic measures**	
Ejection fraction, % (median [IQR)	71 [66.5–73.5]
LVOT MIG at rest, mmHg (median [IQR])	52 [17.5–101]
LVOT MIG with Valsalva, mmHg (median [IQR])	62 [33.5–73.25]
LV mass index, g/m^2^ (median [IQR])	126.5 [103.74–135.5]
Maximum LV wall thickness, mm (median [IQR])	19 [17–21]
LV septal thickness, mm (median [IQR])	15 [13–17]
Left atrial volume index, mL/m^2^ (median [IQR])	46 [35.5–54]

Abbreviations. CKD: chronic kidney disease; Afib: atrial fibrillation; ICD: implantable cardioverter defibrillator; LVOT: left ventricular ejection fraction; MIG: maximum instantaneous gradient.

**Table 2:** Changes in Holter-detected arrhythmias following mavacamten treatment: between baseline and short-term follow-up.

	Baseline *n* = 10	Short-Term *n* = 10	*p*-Value ^1^
**PVC, Median [Q1–Q3]**	24 (2, 286)	19 (7, 494)	0.63
**PAC, Median [Q1–Q3]**	70 (16, 231)	145 (28, 622)	0.69
**Number of SVT runs, Median [Q1–Q3]**	0.25 (0.00, 3.13)	0.76 (0.00, 4.00)	0.67
**SVT max-duration, Median [Q1–Q3]**	2 (0, 10)	6 (0, 19)	0.09
**SVT max-rate, Median [Q1–Q3]**	48 (0, 130)	115 (0, 152)	0.44

Abbreviations. PVC: premature ventricular complex; PAC: premature atrial complex; SVT: supraventricular tachycardiac. ^1^ Wilcoxon singed rank test.

**Table 3:** Changes in Holter-detected arrhythmias following mavacamten treatment: baseline, short-term, and long-term analysis.

	Baseline *n* = 24	Long-Term *n* = 23	*p*-Value ^1^
**PVC, Median [Q1–Q3]**	13 (1, 39)	5 (1, 40)	0.85
**PAC, Median [Q1–Q3]**	75 (24, 166)	53 (18, 204)	0.37
**Number of SVT runs, Median [Q1–Q3]**	0.47 (0.00, 3.00)	1.00 (0.00, 2.00)	0.27
**SVT max-duration, Median [Q1–Q3]**	3 (0, 10)	3 (0, 9)	0.46
**SVT max-rate, Median [Q1–Q3]**	102 (0, 141)	109 (0, 142)	0.9

Abbreviations. PVC: premature ventricular complex; PAC: premature atrial complex; SVT: supraventricular tachycardiac. ^1^ Wilcoxon singed rank test.

## Data Availability

The data that support the findings of this study are available from the corresponding author upon reasonable request, subject to approval by Mayo Clinic’s institutional policies and data use agreements.
